# Interactive Effects between Chronic Lead Exposure and the Homeostatic Iron Regulator Transport *HFE* Polymorphism on the Human Red Blood Cell Mean Corpuscular Volume (MCV)

**DOI:** 10.3390/ijerph16030354

**Published:** 2019-01-27

**Authors:** Chien-Juan Chen, Ting-Yi Lin, Chao-Ling Wang, Chi-Kung Ho, Hung-Yi Chuang, Hsin-Su Yu

**Affiliations:** 1 Graduate Institute of Medicine, College of Medicine, Kaohsiung Medical University, Kaohsiung 80708, Taiwan; jerson.chen@chens.tw (C.-J.C.); yup.kmu@gmail.com (H.-S.Y.); 2 Master Program of Public Health, Kaohsiung Medical University, Kaohsiung 80708, Taiwan; chy@seed.net.tw; 3 Department of Environmental and Occupational Medicine, Kaohsiung Medical University Hospital, Kaohsiung 80708, Taiwan; florawang0913@gmail.com (C.-L.W.); kmco6849@gmail.com (C.-K.H.); 4 Department of Public Health, College of Health Sciences, Kaohsiung Medical University, Kaohsiung 80708, Taiwan

**Keywords:** lead, hemochromatosis, *HFE*, ferritin, mean corpuscular volume MCV

## Abstract

Research has shown that long-term exposure to lead harms the hematological system. The homeostatic iron regulator *HFE* (hemochromatosis) mutation, which has been shown to affect iron absorption and iron overload, is hypothesized to be related to lead intoxication in vulnerable individuals. The aim of our study was to investigate whether the *HFE* genotype modifies the blood lead levels that affect the distributions of serum iron and other red blood cell indices. Overall, 121 lead workers and 117 unexposed age-matched subjects were recruited for the study. The collected data included the blood lead levels, complete blood count, serum iron, total iron binding capacity, transferrin, and ferritin, which were measured during regular physical examinations. All subjects filled out questionnaires that included demographic information, medical history, and alcohol and tobacco consumption. *HFE* genotyping for C282Y and H63D was determined using polymerase chain reaction and restriction fragment length polymorphism (PCR/RFLP). The mean blood lead level in lead workers was 19.75 µg/dL and was 2.86 µg/dL in unexposed subjects. Of 238 subjects, 221 (92.9%) subjects were wild-type (CCHH) for *HFE* C282Y and H63D, and 17 (7.1%) subjects were heterozygous for a H63D mutation (CCHD). Multiple linear regression analysis showed that blood lead was significantly negatively associated with hemoglobin (Hb), mean corpuscular hemoglobin concentration (MCHC), and mean corpuscular volume (MCV), whereas the *HFE* variant was associated negatively with MCV and positively with ferritin. An interactive influence on MCV was identified between blood lead and *HFE* variants. Our research found a significant modifying effect of the *HFE* variant, which possibly affected MCV. The *HFE* H63D heterozygous (CCHD) variant seemed to provide a protective factor against lead toxicity. Future studies should focus on competing binding proteins between iron and lead influenced by gene variation.

## 1. Introduction

Although leaded gasoline has been phased out in many countries in the world, lead continues to be a public health concern due to its widespread industrial uses. Chronic lead exposure has been associated with many adverse health conditions. Recent epidemiological and toxicological studies have reported that lead exposure causes several diseases affecting the cardiovascular system such as hypertension [1] and chronic kidney disease [2,3], as well as the hepatic system [4]. In addition, lead is capable of inducing oxidative stress that damages the brain and affects the life course [5,6], and probably contributes to carcinogenesis [7,8].

Lead has been known to affect the hematological system [9]. However, the detailed mechanism of how lead affects the system remains unclear. Previous literature has documented the possibility of iron deficiency leading to an increase of lead absorption [9,10,11,12]. Lead toxicity to the heme biosynthetic pathway was characterized very well, but the degree of tolerance and thresholds vary among individuals. Regarding the cause, recent research has focused on individual vulnerability, such as gene polymorphisms that could explain the different results arising from the same exposure.

The *HFE* (hemochromatosis) gene, reported about two decades ago [13,14,15]—which was found in patients with hereditary hemochromatosis (HH)—is an autosomal recessive genetic disease producing an increase in the absorption of ingested iron. Affected subjects may develop iron overload, which leads to diabetes, heart disease, and liver disease, but afflicted individuals generally did not present symptoms until mid- to late-adulthood. A homeostatic iron regulator *HFE* gene variant, C282Y, accounts for most cases [16]. H63D, another *HFE* variant, was also associated with hemochromatosis, but has a lower penetrance [15]. Variations in the C282Y gene would change the *HFE* protein structure and mediate increased iron absorption, whereas variations in the H63D gene would interrupt the ability of the *HFE* protein to control an iron-binding protein [16]. Consequently, the *HFE* mutation may result in the functional disturbance of modifying iron absorption [17,18,19]. The combined prevalence of these two polymorphisms may play a role in the general population in both the distribution of body iron and the distribution of any metals that share absorptive pathways with iron, such as lead [10,20,21,22,23]. How the interaction of lead toxicity to heme and *HFE* mutation disturbs iron absorption is still unclear and would be interesting to research.

Previous reports suggested that subjects with clinical HH had higher lead levels or equivalent blood lead levels [24,25,26]. Given that there is a known association between iron absorption and lead absorption, we hypothesized that these genetic variants may be important modifiers of lead toxicodynamics in red blood cells, including mean corpuscular volume (MCV) alterations. A previous study by Onalaja et al. suggested that polymorphisms in the *HFE* gene might influence the absorption of lead, which has not yet been studied, and further studies are needed to identify the effects of genes in lead intoxication [13]. To the best of our knowledge, few studies have examined the toxic effects of lead on the MCV of red blood cells. Therefore, the objective of this study was to investigate whether the *HFE* genotype modifies the blood lead levels that affect the distributions of serum iron and other red blood cell indices.

## 2. Materials and Methods

### 2.1. Subjects

Participants exposed to lead were recruited from a lead recycling manufacturer, where health examinations of workers were carried out annually [27,28]. All lead workers receive an annual health examination according to the Taiwan Occupational Safety and Health Act. Age- and sex-matched workers from the same area but from different plants without lead exposure were selected to serve as the reference group. Their socioeconomic status was similar to that of the lead workers. The exclusion criteria were cancer patients and poor nutrition conditions. We asked the workers whether they agreed to participate in a study that checked their *HFE* genotype (C282Y and H63D) in addition to conducting a regular health examination in 2007, and informed consent forms were obtained before the study. The study protocol was approved by the institutional review board of Kaohsiung Medical University (KMUH-IRB-950305 approved in 2006).

### 2.2. Health Examination

Under the regulation of labor health protections in Taiwan, lead worker health examination should be performed annually. Examinations included physical exams, blood lead tests, hematology tests (including hemoglobin, hematocrit, and complete blood counts), liver function tests, and renal function tests (including serum creatinine and routine urine test). Meanwhile, blood samples were frozen for the analysis of genotypes and other tests permitted by the lead workers and the control group.

### 2.3. Questionnaire

A short questionnaire asking for the workers’ job title, medical and work history, family history, and alcohol and cigarette consumption was administered during the health examination.

### 2.4. Homeostatic iron regulator HFE Genotyping

The *HFE* polymorphisms, both C282Y and H63D, were determined with polymerase chain reaction using restriction fragment length polymorphism (PCR/RFLP) according to the methods described by Cardoso et al. [29]. For C282Y, a DNA sample was amplified with two primers, 5′-TGG CAA GGG TAA ACA GAT CC-3′ and 5′-TAC CTC CTC AGG CAC TCCTC-3′ [16]. The H63D sample was amplified with two primers—5′-ATG GGT GCC TCA GAG CAG-3′ and 5′-AGT CCA GAA GTC AAC AGT-3′—to generate a 210-bp fragment. Joint genotypes were expressed using both categories. For example, a subject that was a wild-type homozygote for both C282Y and H63D was defined as CCHH; a subject homozygous for C282Y and heterozygous for H63D was defined as CCHD.

### 2.5. Statistical Analysis

**Figure 1:**
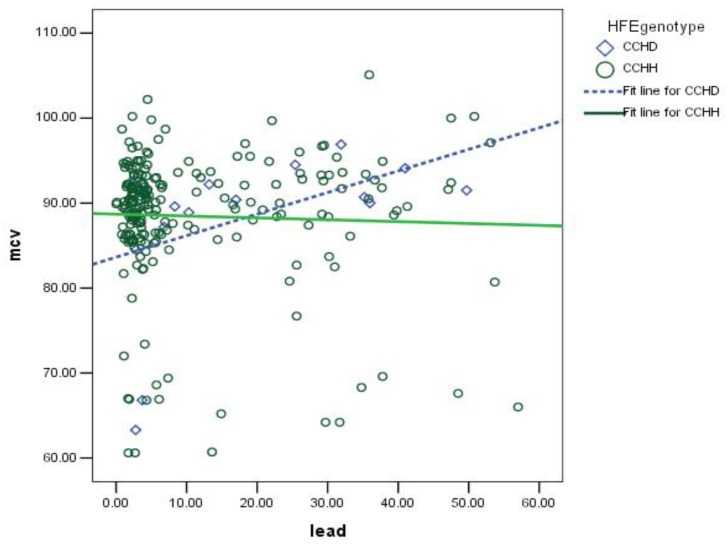
The association between blood lead concentrations (ug/dL) and MCV (fL, or 10^−15^ L) according to different *HFE* genotypes was significant for CCHD but was not significant for CCHH.

We compared the demographic information, blood lead levels, working duration, iron profiles, red blood cell series, and biochemistry data between the lead-exposed and unexposed groups first and then between the CCHH and CCHD genotypes, which were just two types of genotypes that were identified in our subjects. Regression analysis can provide us with the association between dependent variables and contributors by adjusting for potential influencing factors. Therefore, multiple linear regression was employed to analyze the contributions and interactions of blood lead and *HFE* variants to red blood cell series and iron profiles adjusted for age, gender, body mass index (BMI), and smoking and drinking status. Significance was set at 0.05 (two-tailed). To clearly depict the blood lead and MCV according to different *HFE* genotypes, we show the fit line for CCHD and CCHD in Figure 1. All statistical tests were performed using SPSS version 20. Sample size and power were determined using US CDC software Epi-Info prior to the study [30]. Since the difference of blood lead levels between the two groups was large, we set α = 0.05 and power = 0.8. The results showed that the sample size was less than 100 in each group.

## 3. Results

Overall, 121 lead workers and 117 unexposed age-matched subjects were enrolled in the study (Table 1). The mean blood lead levels in the lead workers was 19.75 µg/dL (SD = 14.7), whereas they were only 2.86 µg/dL (SD = 1.9) for unexposed subjects. The mean serum iron in lead workers was 95.7 ± 41.1 µg/dL and was 94.2 ± 40.9 µg/dL in unexposed subjects (*p* = 0.782). Serum iron and iron saturation tended to be higher in lead workers, though it did not reach statistical significance. Lead workers had significantly lower transferrin (230.1 vs. 244.6 µg/dL) and a lower total iron binding capacity (287.6 vs. 305.7 µg/dL) than unexposed workers. In addition, lead workers had a significantly higher creatinine level (1.2 vs. 1.1 mg/dL, *p* < 0.001) compared to unexposed workers, and the lead workers were more likely to use alcohol and tobacco. The number and proportion of CCHH type between the unexposed and lead-exposed groups were 112 (97.5%) and 109 (90.1%), respectively (*p* = 0.15, not significant).

The distribution of *HFE* genotyping in this study showed that all subjects did not have the C282Y mutation (all were CC wild-type) and that 17 workers had the H63D variant (231 homozygote HH type and 17 heterozygote HD type, no DD homozygote). Therefore, of 238 subjects, 221 (92.9%) subjects were CCHH and 17 (7.1%) subjects were CCHD. Table 2 shows a comparison between CCHH and CCHD. Only the number of individuals who smoked were different, and the workers with the CCHD type were more likely to smoke than workers with the CCHH type. The other variables were not significantly different.

Multiple linear regression analysis adjusted for age, gender, BMI, smoking, and drinking status showed that blood lead was significantly associated with hemoglobin (Hb), red blood cells (RBCs), mean corpuscular hemoglobin concentration (MCHC) and MCV, whereas the *HFE* variant (CCHD) was associated with MCV and ferritin. The effect of lead on MCV for the CCHH genotype was 0.116 (standard error (S.E.) = 0.045); whereas the effect of CCHD on MCV was 5.766 (S.E. = 2.827). The interactive influence on MCV was identified between blood lead and *HFE* variant with β = 0.254 (S.E. = 0.125). Table 3 shows 11 regression models; each row was one regression. If the interactions of blood lead and *HFE* variant were not significant, then the regression should omit the interaction term.

To clarify the contribution of lead and *HFE* variants and their interaction, we calculated a fit line for both CCHH and CCHD according to MCV and lead (Figure 1).

## 4. Discussion

Our research shows that there would be a significant modifying effect of lead on a red blood cell series as well as a possible effect of the *HFE* genotype on the absorption and excretion of lead. Lead has been known to cause anemia among workers and children with moderate to high exposure. However, some studies reported that microcytic anemia occurred, whereas others did not. The effect of lead on MCV is still unclear. Our findings suggest that some variability comes from the *HFE* genotypes. The presence of a hemochromatosis variant allele (H63D) predicted lower MCV when the blood lead level increased. Because iron status is inversely associated with lead absorption, we believe that these results may be secondary to increased iron stores among *HFE* variant carriers, which may decrease lead absorption in the gastrointestinal tract.

Hemochromatosis is an autosomal recessive genetic disease with abnormal iron absorption that is more common in Caucasians. Two *HFE* candidate gene mutations are C282Y and H63D. Our results showed no C282Y mutations, and 7.1% of subjects were H63D heterozygotes (there were no homozygotes). In previous studies, 38% of the *HFE* mutations have been reported in Caucasians [31]. Beutler demonstrated 6.3% C282Y mutations and 15.2% H63D mutations in a US population [21]. Another study reported a 12.9% C282Y mutation rate and 25.1% H63D mutation rate in a typical white population [32]. The first report analyzing the prevalence of the *HFE* gene in Mainland China revealed that there were no C282 mutations, and 4.6% of their Han population were H63D heterozygotes [33]. Their report in Mainland China was similar to the results we obtained in Taiwan, and together, these studies suggest that the *HFE* gene mutation may be relatively low in the Han population compared to white populations. A very low prevalence of the *HFE* gene mutation would explain the low prevalence of hemochromatosis in the Han population.

The *HFE* variant may play a role in lead metabolism besides facilitating iron transport and metabolism. Therefore, we hypothesized at the beginning of our research that the functional *HFE* gene may play a role in other metal metabolism, such as lead transport. Our results support our hypothesis that the *HFE* gene polymorphism (H63D in our study) contributes to MCV and the hemopoietic system, especially in red blood cell lines. A review of the literature showed that non-*HFE* genes could predispose individuals to other types of siderosis or hemochromatosis [34]. An investigation by Wright et al. suggested that *HFE* variants influenced lead accumulation after exposure and resulted in lower lead levels, especially for the patella lead level in aging subjects (i.e., an unexposed population) [32].

Lead workers had significantly lower transferrin and a lower total iron binding capacity compared to unexposed workers. We propose that lead more or less combines with iron proteins in the blood in vivo. Previous research by Turgut et al. demonstrated that high copper, cadmium, and lead may decrease iron absorption and negatively affect hematological parameters [35]. Zimmermann et al. suggested that iron fortification reduced blood lead levels in children who had chronic lead exposure [36]. A study by Andrew et al. suggested that iron absorption from the intestine may be transported by a divalent metal transporter (DMT1), which is a hydrophobic protein and mediates the transport of a spectrum of divalent cations. The specificity of DMT1 is not high, and it can transport all other divalent metals, such as zinc, copper, calcium, and lead [37]. This effect may also explain the possible in vivo metal competition. The epidemiological study design may restrict the possibility of direct verification.

## 5. Conclusions

In conclusion, we found evidence that a polymorphism of *HFE* may modify the impact of lead on red blood cells, which is mediated by influencing iron and their binding proteins. With CCHD carriers, there are possible protective effects at certain levels of lead burden compared to CCHH individuals. To our knowledge, this study may be the first attempt to clarify the effects of lead exposure on hematological systems, especially in red blood cells influenced by *HFE gene* variations in Taiwan. Additional research is needed to determine if this gene–environment interaction has clinical significance in particular Asian groups.

## Figures and Tables

**Table 1:** Comparison between the unexposed group and the lead-exposed group.

Characteristics	Non-Exposure *N* = 117	Lead Exposure *N* = 121	*p*-Value *
Age (years)	41.7 ± 11.8	41.5 ± 8.3	0.917
Gender, male (%)	71 (60.7)	98 (81.0)	0.001
Body mass index (BMI)	24.9 ± 4.1	24.4±3.5	0.310
Working duration (year)	--	11.8 ± 5.3	
Blood lead (µg/dL)	2.9 ± 1.9	19.8 ± 14.7	<0.001
Serum iron (µg/dL)	94.2 ± 40.9	95.7 ± 41.1	0.782
Iron saturation (%)	31.7 ± 15.1	33.9 ± 15.1	0.272
Ferritin (ng/mL)	210.3 ± 280.3	183.2 ± 162.8	0.367
Transferrin (µg/dL)	244.6 ± 34.4	230.1 ± 35.2	0.002
TIBC (µg/dL)	305.7 ± 43.0	287.6 ± 44.0	0.002
Hemoglobin (Hb) (g/dL)	14.7 ± 1.5	15.0 ± 1.4	0.168
Red blood cells (RBCs) (×10^6^/μL)	4.9 ± 0.6	5.1 ± 0.6	0.057
Mean corpuscular volume (MCV) (fL)	88.5 ± 7.5	88.4 ± 8.4	0.957
Creatinine (mg/dL)	1.1 ± 0.2	1.2 ± 0.2	<0.001
AST (IU/L)	17.0 ± 11.1	19.2 ± 9.9	0.114
ALT (IU/L)	18.4 ± 16.0	21.6 ± 21.3	0.203
Smoking, yes (%)	16 (13.7)	49 (40.5)	<0.001
Drinking, yes (%)	9 (7.7)	32 (26.4)	<0.001
*HFE* genotype			
CCHH (%)	112 (97.5)	109 (90.1)	0.150
CCHD (%)	5 (4.3)	12 (9.9)	

* Chi-square or *t*-test. CCHH: subject wild-type homozygous for both C282Y and H63D; CCHD: subject homozygous for C282Y and heterozygous for H63D. TIBC—Total iron-binding capacity; AST—aspartate transaminase; ALT—alanine transaminase.

**Table 2:** Comparison between the CCHH and CCHD genotypes.

Characteristics	CCHH (n = 221)	CCHD (n = 17)	*p*-Value *
Age (years)	41.4 ± 10.2	44.6 ± 9.4	0.208
Gender, male (%)	154 (69.7%)	15 (88.2%)	0.163
BMI	24.6 ± 3.8	25.4 ± 3.5	0.377
Lead exposure, n (%)	109 (49.3%)	12 (70.6%)	0.13
Blood lead (µg/dL)	11.0 ± 13.3	17.2 ± 15.9	0.067
Serum iron (µg/dL)	94.2 ± 41.2	104.9 ± 36.7	0.296
Iron saturation (%)	32.5 ± 15.1	37.2 ± 15.5	0.218
Ferritin (ng/mL)	185.6 ± 213.8	337.6 ± 343.1	0.090
TIBC (µg/dL)	296.7 ± 44.4	292.8 ± 45.1	0.723
Transferrin (µg/dL)	237.4 ± 35.5	234.2 ± 36.1	0.723
RBC (×10^6^/μL)	5.0 ± 0.6	5.1 ± 0.4	0.536
Hb (g/dL)	14.8 ± 1.4	15.2 ± 1.5	0.339
MCV (fL)	88.5 ± 7.9	88.0 ± 9.1	0.826
Creatinine (mg/dL)	1.2 ± 0.2	1.1 ± 0.1	0.188
AST (IU/L)	18.4 ± 10.9	15.8 ± 2.8	0.343
ALT (IU/L)	20.4 ± 19.6	17.5 ± 9.4	0.558
Smoking, yes (%)	56 (25.3%)	9 (52.9%)	0.022
Drinking, yes (%)	36 (16.3%)	5 (29.4%)	0.183

* Chi-square or *t*-test. MCV— mean corpuscular volume; BMI—body mass index.

**Table 3:** The contributions and interactions of blood lead and the *HFE* variant, which were determined by a multiple linear regression model adjusted for age, BMI, and smoking and drinking status.

Contributions/ Dependent Variables	Blood Lead (µg/dL)	*HFE* Variant (CCHD)	Blood Lead and CCHD Interaction
Hb	−0.014 (0.006) *	−0.035 (0.276)	NS
RBC	0.006 (0.003) *	0.047 (0.136)	NS
Hct	−0.019 (0.016)	−0.475 (0.707)	NS
MCH	−0.036 (0.020)	−0.207 (0.869)	NS
MCHC	−0.016 (0.005) *	0.267 (0.233)	NS
MCV	−0.116 (0.045) *	−5.766 (2.827) *	0.254 (0.125) *
Serum iron	0.165 (0.233)	7.964 (10.332)	NS
Ferritin	−1.977 (1.235)	121.411 (54.701) *	NS
TIBC	−0.313 (0.252)	−2.833 (11.170)	NS
Transferrin	−0.251 (0.202)	−2.265 (8.936)	NS
Iron saturation	0.086 (0.085)	3.588 (3.772)	NS

Presented by β (s.e.). All measures were adjusted for age, gender, BMI, smoking, and drinking status. MCHC—mean corpuscular hemoglobin concentration; NS—not significant; Hct—hematocrit; MCH—mean corpuscular hemoglobin. * *p* < 0.05.
